# Re-administration of SmallPox vaccine against MonkeyPox among general population; after more 30 Years of vaccine cessation by WHO

**DOI:** 10.1016/j.amsu.2022.104519

**Published:** 2022-09-06

**Authors:** Sandhaya Kukreja, Sidhant Ochani, Asifa Kalwar, Sapna Lohana, Kaleem Ullah

**Affiliations:** a Dow University of Health and Sciences, Karachi, Pakistan; b Khairpur Medical College, Khaipur Mir's, Pakistan; c Pir Abdul Qadir Shah Jeelani Institute of Medical Sciences, Gambat, Pakistan

## Abstract

Monkeypox virus, Orthopoxvirus of the Poxviridae family which was first isolated from pox lesions of monkeys, hence named monkeypox**.** Fever, rash, and enlarged lymph nodes are common symptoms, but it can also lead to a variety of medical complications, including secondary infections, bronchopneumonia, sepsis, encephalitis, and corneal infection with vision loss. The current outbreak doesn't follow previous pattern of transmission through animals but person to person transmission. We highlight several studies including WHO stats for protective role of small pox vaccine and recommend potential strategies for current and future disease eliminations.

Respected Editor,

The monkeypox virus, belongs to the Orthopoxvirus of the Poxviridae family which was first isolated from pox lesions of monkeys in 1958, hence named monkeypox**.** The first human affected by this virus was a 9-year-old child in the Democratic Republic of the Congo in 1970**.** Since then, occasional occurrences of human monkeypox have been documented, especially in Central and West Africa [1]. Following the transportation of infected animals from Ghana to Texas, the first monkeypox outbreak outside of Africa was discovered in the United States in 2003 [2]. The virus has been divided into two genetically distinct clades: the Congo Basin (Central African) clade and the West African clade. The Congo Basin is more dangerous than the West African clade, with a case fatality rate of 10.6% against 3.6% for the West African clade [3]. Monkeypox takes 6–13 days to incubate, although it can take anywhere from 5 to 21 days. Monkeypox has a similar clinical presentation to smallpox, however it is less severe and contagious. Fever, rash, and enlarged lymph nodes are common symptoms, but it can also lead to a variety of medical complications, including secondary infections, bronchopneumonia, sepsis, encephalitis, and corneal infection with vision loss. Rope squirrels, tree squirrels, Gambian pouched rats, dormice, non-human primates, and other species are natural hosts. The virus spreads through close face-to-face, skin-to-skin, mouth-to-mouth or mouth-to-skin contact, including sexual contact. There is also an evidence of transmission through fomites, short range aerosols and during childbirth through close contact [4].

The current outbreak, which has been confirmed or suspected in the United States, the United Kingdom, Spain, Portugal, Italy, Belgium, Sweden, France, Canada, Australia, Germany, and the Netherlands, does not appear to have followed the same pattern as previous outbreaks outside of Africa, which were almost all linked to importation via flights from Africa or exposure to infected exotic pets. What makes these new cases unique, all of which occur outside of the virus's endemic region—is that they involve person-to-person transmission [5]. Many of the cases reported have been found in men who have sex with other men. Monkey pox rashes can resemble several sexually transmitted diseases, such as herpes and syphilis, which could help to explain why these cases are frequently discovered in homosexual or bisexual communities at sexual health clinics and reflect this population's proactive approach to health [4]. Smallpox is clinically and immunologically similar to monkeypox. By the end of 1979, the widespread use of a vaccinia virus-based vaccination in at-risk communities had effectively stopped the spread of smallpox, resulting in the worldwide elimination of the naturally occurring illness [6]. It has been more than 30 years since the World Health Organisation (WHO) recommended that routine smallpox vaccination be discontinued. Few people born following the eradication of smallpox have gotten vaccination, and immunity is waning among those who did. The decline in population immunity associated with discontinuation of smallpox vaccination may have set the stage for a resurgence of monkeypox [7]. This is evidenced by an increase in the number of monkeypox cases as well as a resurgence of the disease in some countries after an absence of 30–40 years [3]. In a study of confirmed and suspected cases of monkeypox in the CAR, 19.2% (5/26) had a smallpox vaccination scar, and the overall attack rate was lower among vaccinated individuals (0.95/1000) compared to unvaccinated individuals (3.6/1000) [8]. On July 23, the World Health Organisation declared monkeypox outbreak a public health emergency of international concern. The Strategic National Stockpile (SNS) stated that they contained adequate smallpox vaccine for administration, however after the declaration that created a sense of panic amidst COVID, tremendous amount of people rushed for vaccinations and vaccines are now limited and out of stock. Additionally, a monkeypox specific vaccine named “JYNNEOS” has been developed which is the only FDA-licensed vaccine in the United States that is approved for prevention, but only 2400 doses were available in the SNS at the start of the outbreak [9].

Previous literature demonstrates smallpox vaccine as 85% protective against monkeypox. Therefore we recommend re-administration of vaccines to general population, especially people at risk i. e; healthcare workers and patients with contact history with suspected or confirmed cases. Hand washing, maintaining good hygiene and using protective equipments should be adapted as a part of routine. Additionally, spreading awareness among homosexual and bisexual communities about the risk and protective measures. Looking back to historical outbreaks we conclude that Africa is a hub for infectious diseases, therefore targeting the roots as a potential strategy in eliminating several diseases and risk of another outbreak of Disease X.

## Ethical approval

Not Applicable.

## Sources of funding

The author(s) received no financial support for the research, authorship, and/or publication of this article.

## Author contribution

**SK** conceptualized the topic**. SO** did literature review. **SK**, **AK**, &**SL** wrote the manuscript. **SO** and **KU** did review-editing and referencing.

## Registration of research studies

Not Applicable.

## Guarantor

All authors take responsibility for the work, access to data and decision to publish.

## Consent

Not Applicable.

## Declaration of competing interest

The author(s) declared no potential conflicts of interest concerning the research, authorship, and/or publication of this article.
